# COVID-19 Knowledge Resource Categorization and Tracking: Conceptual Framework Study

**DOI:** 10.2196/29730

**Published:** 2021-06-01

**Authors:** Muhammad Afzal, Maqbool Hussain, Jamil Hussain, Jaehun Bang, Sungyoung Lee

**Affiliations:** 1 Department of Software Sejong University 209 Neungdong-ro Seoul Republic of Korea; 2 Department of Data Science Sejong University Seoul Republic of Korea; 3 Department of Computer Science and Engineering Kyung Hee University Yongin Republic of Korea

**Keywords:** information organization, resource management, knowledge graphs, interactive dashboard, dependency tracking, COVID-19, pandemic, information technology, tracing information, dashboards, digital health

## Abstract

**Background:**

Since the declaration of COVID-19 as a global pandemic by the World Health Organization, the disease has gained momentum with every passing day. Various private and government sectors of different countries allocated funding for research in multiple capacities. A significant portion of efforts has been devoted to information technology and service infrastructure development, including research on developing intelligent models and techniques for alerts, monitoring, early diagnosis, prevention, and other relevant services. As a result, many information resources have been created globally and are available for use. However, a defined structure to organize these resources into categories based on the nature and origin of the data is lacking.

**Objective:**

This study aims to organize COVID-19 information resources into a well-defined structure to facilitate the easy identification of a resource, tracking information workflows, and to provide a guide for a contextual dashboard design and development.

**Methods:**

A sequence of action research was performed that involved a review of COVID-19 efforts and initiatives on a global scale during the year 2020. Data were collected according to the defined structure of primary, secondary, and tertiary categories. Various techniques for descriptive statistical analysis were employed to gain insights into the data to help develop a conceptual framework to organize resources and track interactions between different resources.

**Results:**

Investigating diverse information at the primary, secondary, and tertiary levels enabled us to develop a conceptual framework for COVID-19–related efforts and initiatives. The framework of resource categorization provides a gateway to access global initiatives with enriched metadata, and assists users in tracking the workflow of tertiary, secondary, and primary resources with relationships between various fragments of information. The results demonstrated mapping initiatives at the tertiary level to secondary level and then to the primary level to reach firsthand data, research, and trials.

**Conclusions:**

Adopting the proposed three-level structure allows for a consistent organization and management of existing COVID-19 knowledge resources and provides a roadmap for classifying future resources. This study is one of the earliest studies to introduce an infrastructure for locating and placing the right information at the right place. By implementing the proposed framework according to the stated guidelines, this study allows for the development of applications such as interactive dashboards to facilitate the contextual identification and tracking of interdependent COVID-19 knowledge resources.

## Introduction

The novel coronavirus—SARS-CoV-2—first appeared in December 2019 and has quickly spread over other regions of the world. The World Health Organization (WHO) declared COVID-19, the disease caused by SARS-CoV-2, a global pandemic [1,2]. It gained momentum as every day passed, and private and government sectors of different countries pushed funding toward research on COVID-19 in various capacities. A portion was dedicated to investing in vaccine discovery and personal protective equipment manufacturing. The other portion was devoted to information technology and service infrastructure development, including research to develop intelligent models and techniques for alerts, diagnosis, treatment, prognosis, prevention, and other relevant services [3]. Our study focused on the second part of the research initiatives related to COVID-19 and provides a comprehensive review of such initiatives.

A rapid and timely response was required from the world to circumvent the challenge of COVID-19. Various organizations from different countries concentrated on investigations and discoveries in data, information, and knowledge to support population health [4]. We designed a structure for categorizing efforts and initiatives related to COVID-19 into three levels or categories: primary, secondary, and tertiary. The primary level represents resources and initiatives that accrue raw data or research about patients with COVID-19 and bring it into the global space. The secondary level encompasses resources and initiatives that analyze primary-level resources, making it more meaningful by adding metadata and filtering out unnecessary items in the data. The tertiary level includes resources and initiatives that consolidate the first- and second-level efforts by creating guidelines, code systems, standard resources, and vocabularies. This study provides a review of initiatives globally at these three levels and proposes a novel research dashboard for tracking COVID-19 resources with dependency workflows. The proposed dashboard will provide a gateway to global initiatives with enriched contextual metadata to help users track the information flow at different levels for validation and verification.

This study is the first of its kind to categorize efforts and initiatives in the form of resources related to COVID-19. It was inspired from the research sources outlined in commentaries and library handouts we consulted [5,6]. This study improvises the three types of information sources to map COVID-19–related initiatives to each kind and devises a method of finding their interdependence for easy tracking of information in contextual trails and metadata. In the context of COVID-19, we can find studies that discuss data sets and techniques, but no such research has been found to categorize initiatives and their dependencies.

## Methods

### Review of COVID-19 Efforts and Initiatives

**Figure 1:**
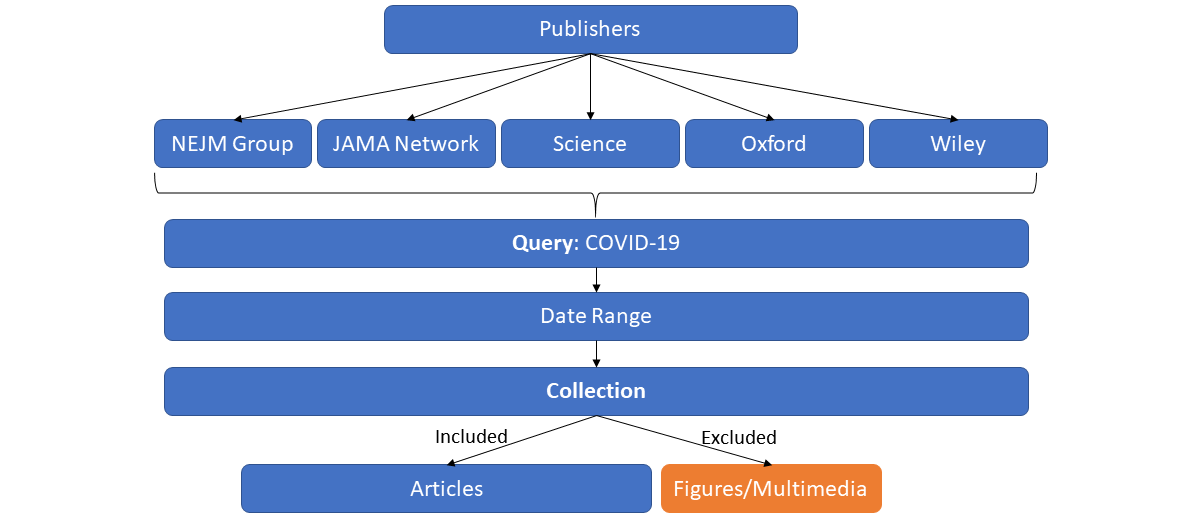
Publisher-wise searching for published research articles on COVID-19. NEJM: New England Journal of Medicine, JAMA: Journal of the American Medical Association.

This study explored initiatives through an informal search strategy to identify key initiatives at each level; however, the authors do not claim the list to be exhaustive. The authors’ experience in evidence-based medicine and translational research made it easy to reach out to well-known data resources and look for COVID-19–related efforts. The study did not distinguish the type of data and information pertinent to a specific initiative; instead, it focused on resources. For different resources, different search strategies were employed. For example, primary-level studies were searched in top publisher networks by using the procedure described in Figure 1. For secondary- and tertiary-level resources, a mixed method of free searching, word of mouth, and expert recommendations were used.

### Research Design

This research reports on phase 1 of an action research project. We designed a conceptual framework for categorizing COVID-19 resources and surveyed the efforts and initiatives that contributed to creating these resources. This first phase establishes the infrastructure for locating and placing the right information at the right place. It provides a schema guideline for developing implementation models and systems.

Phase 2 involves the development of a software system and testing the viability of the proposed approach by undertaking the software development process model for each component and deploying on to an open repository for global access.

**Figure 2:**
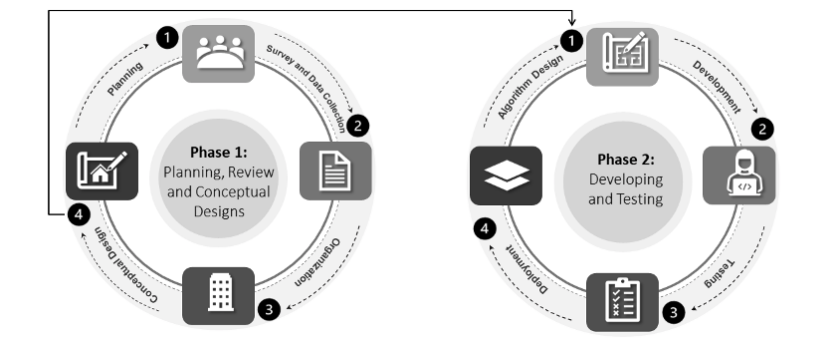
Action research phases.

Figure 2 provides an overview of the two phases of the action research design. Planning includes brainstorming sessions, informal and formal meetings, and surveying COVID-19 efforts and initiatives to collect data. The initial data were organized to get a sense of the data elements through visuals that included bar graphs, pie charts, and histograms. Conceptual design is the design of an architecture framework for the categorization of resources. Algorithm design involves knowledge graphs, data management and querying, data-driven approaches, and user interface and user experience. Development is the actual implementation of the designed methods in the chosen programming languages. Deployment is the reflection and availability of developed methods and models to the community via open-access platforms like GitHub. As described, at any stage of phase 2, the process of phase 1 can be accessed and replicated.

## Results

### COVID-19 Efforts and Initiatives

#### Primary Resources

The primary level includes resources that accumulate raw data or research about patients with COVID-19 and bring it into the global space. Primary-level resources were categorized into three groups: research literature, data, and clinical trials. Research literature includes published information like journal publications, raw data includes firsthand patient information like demographic and clinical information, and clinical trials includes registered trials with the objective to prove the effectiveness of a treatment like a drug or surgery. We provided a statistical overview of different resources in each group.

#### Research Literature

The research literature group was divided into two categories: preprint and postprint. Preprint articles are “work in progress” research that are yet to be published in a peer-reviewed journal. Postprint articles are “submitted to journal” research that are considered published after going through copyediting and typeset formatting.

### Preprint Literature

Alongside published research literature, many research articles were submitted to preprint repositories, which are unreviewed. They may eventually be published in a peer-reviewed journal. Due to unpredictable review durations, most researchers first submit an earlier version of their paper to a preprint database to disseminate their work quickly. Among popular preprints, arXiv [7] is an extensive multidisciplinary archive with 1.7 million scholarly articles in physics, mathematics, computer science, quantitative biology, quantitative finance, statistics, electrical engineering and systems science, and economics. It published 2842 articles on COVID-19 in 2020. Related to the medical and biology domains, two archives—medRxiv and bioRxiv [8]—are at the forefront in the collection unreviewed literature. As of 2020, medRxiv and bioRxiv published 9487 and 3058 articles, respectively. Other archives like ChemRxiv, a preprints server for chemistry and related areas, has also published a considerable number of prereviewed articles on COVID-19 (n=364).

### Postprint (Published) Literature

**Figure 3:**
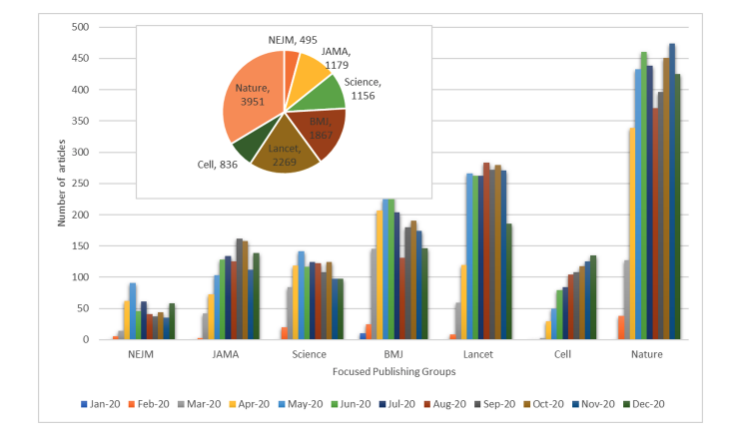
Monthly progression of publications on COVID-19 according to publisher. NEJM: New England Journal of Medicine, JAMA: Journal of the American Medical Association.

At present, a vast number of publications in the biomedical literature mention “COVID-19” in the title, the body of text, the keywords, or metadata. From January 1 to December 31, 2020, about 12,000 articles were published by prominent publishers that include Nature, Science, Cell, the New England Journal of Medicine (NEJM), the Journal of the American Medical Association (JAMA), Lancet, and BMJ. As shown in Figure 3, Nature published a total of 3951 articles, contributing about 34%, followed by Lancet (19%), in the given group of 7 publishers. It should be noted that this quantitative representation does not discuss the quality of articles. Later in this study, we will discuss COVID-19–relevant and COVID-19–specific articles. Article count increased every month initially, with the maximum number of articles appearing between May and October 2020. Starting in January 2020, there were hardly any substantive articles specific to COVID-19, although the count increased to a two-digit number in February. A maximum number was observed in October with 1364 articles. Articles continue to be published at a steady rate.

**Figure 4:**
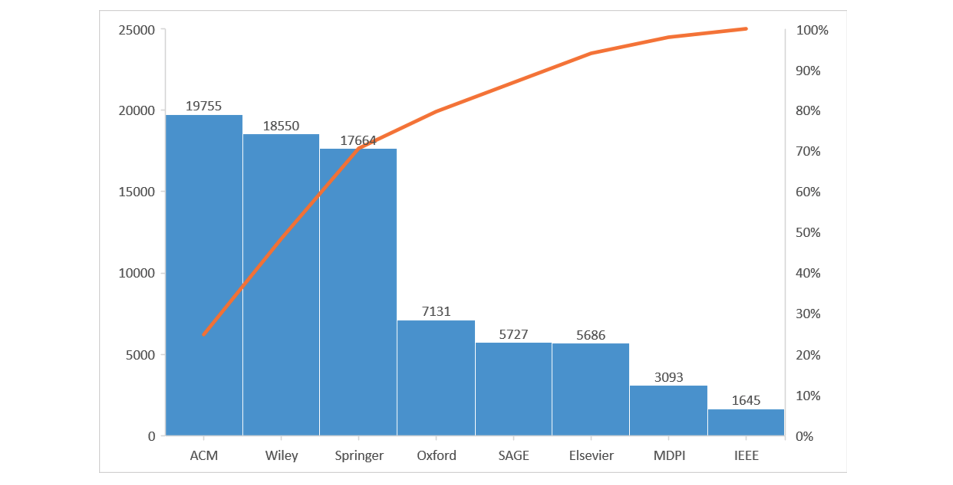
Number of articles that appeared in major scientific libraries accessible to a researcher, presented in decreasing order from January to December 2020. The orange line displays the cumulative %, demonstrating that only three major scientific libraries (ACM, Wiley, and Springer) contributed about 70% of the articles. ACM: Association for Computing Machinery.

Many articles are being added to major libraries daily. We surveyed 8 major scientific libraries: Association for Computing Machinery (ACM), Wiley, Springer, Oxford, SAGE, Elsevier, MDPI, and IEEE. All these databases are accessible to researchers; therefore, we opted to present their statistics. We used the term “COVID-19” to keep the search simple, with increased coverage and reduced chances of missing COVID-19–related publications. As shown in Figure 4, ACM alone identified about 20,000 entries on COVID-19–related articles. Figure 4 indicates that COVID-19–related articles are published everywhere, including in IEEE Explore even though its focus is more on engineering.

#### Patient Data Sets

Data play a crucial role in the ability to research, study, and explore population health and safety, and this is truer than ever in the context of a global pandemic. Access to data sets and associated tools that can examine that data are increasingly crucial to the research process and are particularly necessary for the worldwide response to the novel coronavirus.

To aid researchers, developers, and analysts in the struggle to fight COVID-19, different platforms offer data sets about patients with COVID-19 like the Johns Hopkins Center for Systems Science and Engineering (CSSE) [9], the Google COVID-19 Public Dataset Program [10], Kaggle [11], and GitHub [12]. Only on Kaggle are there 972 data sets of small (n=773), medium (n=168), and large (n=24) sizes. Most of these data sets are available in CSV (comma-separated value) or MS Excel (Microsoft Corp) formats. On GitHub, which is primarily a repository for hosting project code files developed in different languages, such as Python, data are also stored for the code reproducibility alongside the code files. As of June 10, 2020, 4073 public repositories have been added to GitHub matching the topic of COVID-19. Several other resources, including data.world [13], AMiner [14], and IEEEDataPort [15], provide free data sets related to COVID-19. Hundreds of regional and country-specific data sets are available through different channels; however, it is not within the scope of this study to describe them here. Rather, this study aims to present platforms that host data sets across regions and countries.

For public health safety and security, a few global-level information resources such as the WHO [16] and Centers for Disease Control and Prevention (CDC) [17] are worthy of mention as they provide important information for public awareness. The WHO is a global entity that accumulates information from its member organizations and disseminates it on its website and through other public interest channels. The WHO developed a specialized tool called the Coronavirus Disease (COVID-19) Dashboard [9], which provides a rich spectrum of metadata and analytics about COVID-19.

Whether in the United States or abroad, the CDC fights diseases that are chronic or acute, curable or preventable, and caused by human error or deliberate attack. It is a rich source of information on COVID-19 in terms of symptoms, risk factors, and guidelines on social distancing, and provides important information to different stakeholders like policymakers, travelers, businesspeople, schools, health professionals, the general public, and high-risk populations. To facilitate users’ understanding of the nature of data, we describe popular data set platforms in Table 1, reporting characteristics helpful for developing statistical and machine learning models for further analysis and research. In addition to defining the platform’s aim and scope, we also provide a brief explanation of the nature of the data sets hosted on that platform to help users spend their time appropriately. Some platforms offer data that can be used solely for statistical analysis and reporting purposes. In contrast, others could be used for assistance in clinical decision making related to patient diagnosis, treatment, and prognosis. In Table 2, we provide summarized information about data resources on COVID-19.

**Table 1:** A brief description of COVID-19 platforms that offer an open repository of data sets and their scope and usefulness.

Data set platform	Description	COVID-19 data sets/repositories, n
Kaggle [11]	Kaggle is the world’s largest data science community with powerful tools and resources to achieve data science goals.	972 data sets
GitHub [12]	GitHub is primarily a host for software development and version control; however, alongside the code files, the associated data are also available for code reproducibility.	4073 repositories
data.world [13]	data.world is an open data resource hub on COVID-19 with contributions from thousands of users and organizations worldwide.	40 data sets
AMiner [14]	AMiner collects all kinds of data sets about COVID-19 with daily updates. The data are open and available for download.	438 data sets
IEEEDataPort [15]	IEEEDataPort provides free data set storage and hosts different types of data sets. It provides space to host data sets related to COVID-19. It hosts a large set of tweets data (n=174,573,543) in the English language from around the globe.	15 data sets
Google COVID-19 Public Dataset Program [10]	To make data more accessible to researchers, data scientists, and analysts, Google created a COVID-19 Public Datasets Program that hosts a repository of public data sets and is free to access and analyze.	3 data sets (JHU CSSE^a^ data set, global health data from the World Bank, and OpenStreetMap data)

^a^JHU CSSE: Johns Hopkins University Center for Systems Science and Engineering.

**Table 2:** A brief description of COVID-19–related information resources.

Organization	Description	Key information services
World Health Organization (WHO) [18]	The WHO works with 194 member states across 6 regions and from more than 150 offices, striving to combat diseases—communicable diseases like influenza and HIV, and noncommunicable diseases like cancer and heart disease.	A COVID-19 dashboard that provides up-to-date case information, including the number of deaths and recovery Generates advice for public awareness to reduce the chances of being infected or spreading COVID-19 Situation reports, released daily, provide the current COVID-19 epidemiological situation and present official case and death counts and transmission classifications. As of June 16, 2020, 148 situation reports have been released Other services include travel advice, training and exercise, technical guidance, response funds, etc
Centers for Disease Control and Prevention (CDC) [17]	The CDC is a US-based organization that aims to provide information on health safety and security threats, both foreign and domestic.	Information on symptoms, risk factors, and social distancing A bank of answers to important questions and many guidance materials for various stakeholders including travelers, health care professionals, etc
EU^a^ Open Data Portal [19]	The European CDC publishes a data set that contains the latest available public data on COVID-19 worldwide by screening up to 500 relevant sources every day between 6:00 and 10:00 CET.	COVID-19 cases worldwide for download Visualizations of cases geographically, situation dashboard, and other graphical representations Documentation as to how the data are collected, a script of R software, and webinars
Johns Hopkins University (JHU) COVID-19 Dashboard [9]	JHU experts designed a rich and interactive COVID-19 dashboard to inform the public, help policymakers create awareness, and save lives.	An interactive dashboard for tracking global COVID-19 cases Animated maps that show total cases, deaths, and new cases Critical trends on how the novel coronavirus is spreading around the globe Worldwide mortality analysis
The World Bank COVID-19 case data [20]	The World Bank provides an array of real-time data, statistical indicators, and other types of data relevant to COVID-19, particularly on the economic and social impacts of the pandemic and the World Bank’s efforts to address them.	Global poverty estimates of the impact of COVID-19 Health nutrition and population statistics Understanding the COVID-19 pandemic through data on indicators and worldwide cases Map of the World Bank’s operational response to the coronavirus and relevant services
DXY [21]	DXY provides timely, accurate, and authoritative real-time reports on the COVID-19 pandemic through global mapping and knowledge.	Global mapping of coronavirus cases COVID-19 knowledge for the public, doctors, etc
National Institutes of Health (NIH) COVID-19 Research [22]	The NIH offers a specialized service that provides the latest research information on COVID-19.	ACTIV (Accelerating COVID-19 Therapeutic Interventions and Vaccines) Treatment guidelines Grants and funding information COVID-19 testing information

^a^EU: European Union.

#### Clinical Trials

Clinical trials are conducted to evaluate the effectiveness and safety of medications or medical devices by monitoring their effects on a select population. Clinical trials pass through two stages: registration and publishing. Preferably, every trial should have at least one results article, even if the results are not significant or produce negative findings; however, sometimes it may be harder to publish due to publication bias [23]. A trial can be linked to a journal article through an unstructured trial-article link (may not involve unique identifiers) or a structured trial-article link (a computable link assigned with unique identifiers such as the ClinicalTrials.gov ID or the PubMed ID) [24].

Several platforms are functional such as those that register primary clinical trials related to COVID-19 and are freely accessible and searchable. The WHO International Clinical Trials Registry Platform (ICTRP) [25] was established to facilitate a network of international clinical trial registers to ensure a single point of access, the unambiguous identification of trials, and public accessibility. From January 2020 to June 2020, a total of 3163 clinical trials have been registered in the WHO ICTRP database from 18 different sources that include ClinicalTrials.gov; Chinese Clinical Trials Registry (chiCTR); Australia New Zealand Clinical Trial Registry; Clinical Research Information Service, Republic of Korea; Clinical Trial Registry–India; EU Clinical Trials Register; German Clinical Trials Register; Iranian Registry of Clinical Trials; International Standard Randomised Controlled Trial Number Registry (ISRCTN); Japan Primary Registries Network; Lebanese Clinical Trial Registry; Netherlands Trial Register; Pan African Clinical Trials Registry; Brazilian Clinical Trials Registry; Peruvian Clinical Trial Registry; Cuban Public Registry of Clinical Trials; Sri Lanka Clinical Trials Registry; and Thai Clinical Trials Registry.

The US-based ClinicalTrials.gov [26] is perhaps the largest database of privately and publicly funded clinical studies conducted worldwide. In this database, we found a total of 2172 studies related to COVID-19 as of June 18, 2020. The second largest clinical trials database is the chiCTR [27], a nonprofit Chinese platform that has registered 721 clinical trials. All the registries are region or country specific except the ISRCTN Registry, which initially focused on randomized controlled trials; however, but has now widened its scope to include other study types to evaluate the efficacy of human-related health interventions [28]. As of June 18, 2020, the ISRCTN database registered 70 clinical trials, out of which only 6 have been completed, 4 have been suspended, and 60 are in progress.

**Figure 5:**
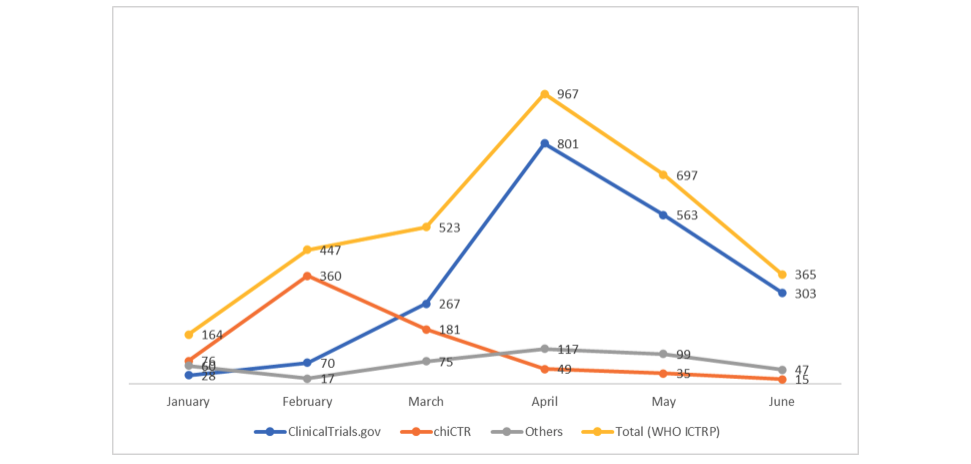
Progression of the number of trials registered in major databases between January and June 2020. chiCTR: Chinese Clinical Trials Registry, WHO ICTRP: World Health Organization International Clinical Trials Registry Platform.

Figure 5 shows a progress bar of trials registered in the first 6 months of 2020. The highest number of trials (n=967) had been registered in April. The number started declining after April, which was generally seen across all databases except the chiCTR, where the decline began after February. This may be because, in China, the peak number of COVID-19 cases was observed in February. The number of trials in May and June should be higher than in April as the number of positive cases continues to rise globally.

**Figure 6:**
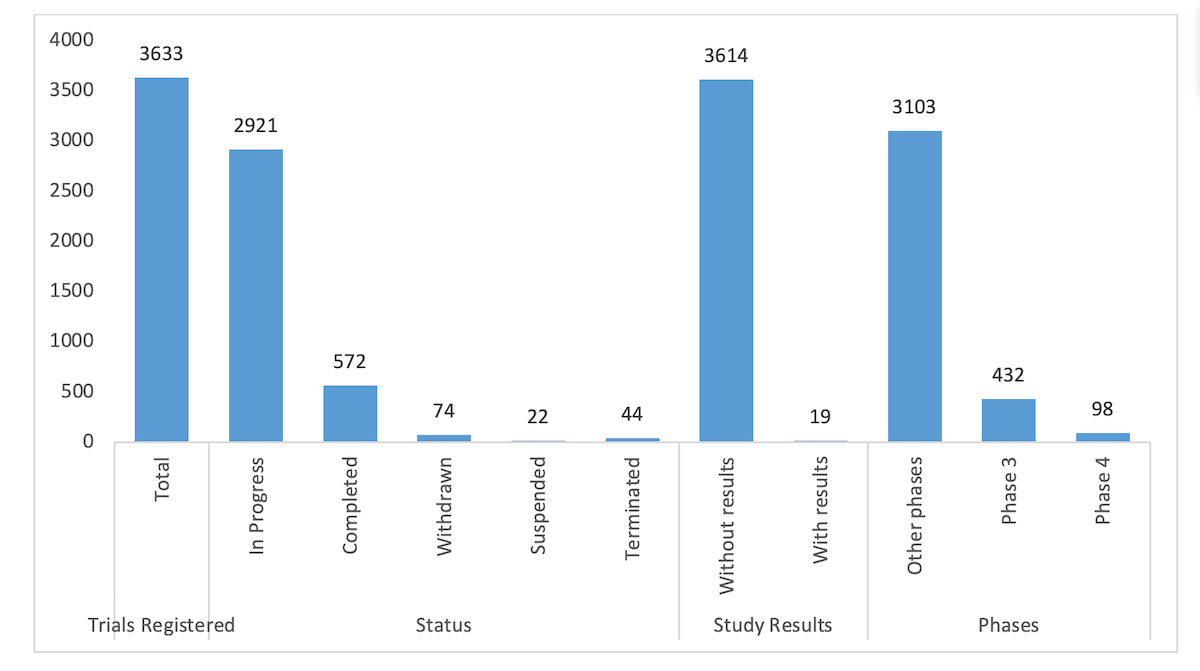
Statistical summary of clinical trials registered in the ClinicalTrials.gov database according to the trial status (eg, completed, withdrawn, suspended, terminated, or in progress), trials with results, and trials registered in different phases.

Figure 6 provides a statistical summary of ClinicalTrials.gov that includes the number of trials in 2020. The data show that only 572 trials have been completed, out of which only 19 have been reported with results. Phase IV, also called a postmarketing surveillance trial, occurs after the Food and Drug Administration has approved a drug for marketing and is the only trial that is eligible for observing drug use in public. At present, the number of phase IV trials (n=98) is far less than the phase III trials (n=432) and other trials in other phases. However, it is a reasonable number to raise the hope of obtaining significant results from watching the effects of drug on a large number of patients with COVID-19 around the world.

#### Secondary Resources

In this section, we present global-scale initiatives founded on the data acquired from primary-level sources at the secondary level. These initiatives are not discussed in any specific order. Most of these initiatives were pertinent to creating metadata on research literature articles for subsequent analysis and research.

#### CORD-19

CORD-19 (COVID-19 Open Research Dataset) is a free, open research data resource consisting of 130,000 scholarly articles about the novel coronavirus available for the global research community [29]. CORD-19 is updated every week with newly published research to facilitate the development of text mining and information retrieval systems, and it has been downloaded over 75,000 times in the first month of its release [29]. The articles in CORD-19 are derived from four primary-level repositories that include PubMed Central (PMC) [30], the bioRxiv and medRxiv preprint servers, and the WHO COVID-19 Database. The significant accomplishment of CORD-19 is the cleaning of metadata and machine readability of the full text. A simple deduplication logic of creating clusters for retaining similar articles, unless there is a conflict, is applied after metadata from each source is cleaned and formatted into CORD-19. After cleaning, the content is parsed from PDF-formatted papers into a JSON (JavaScript Object Notation) schema, which is simple to utilize for different text-mining tasks.

**Figure 7:**
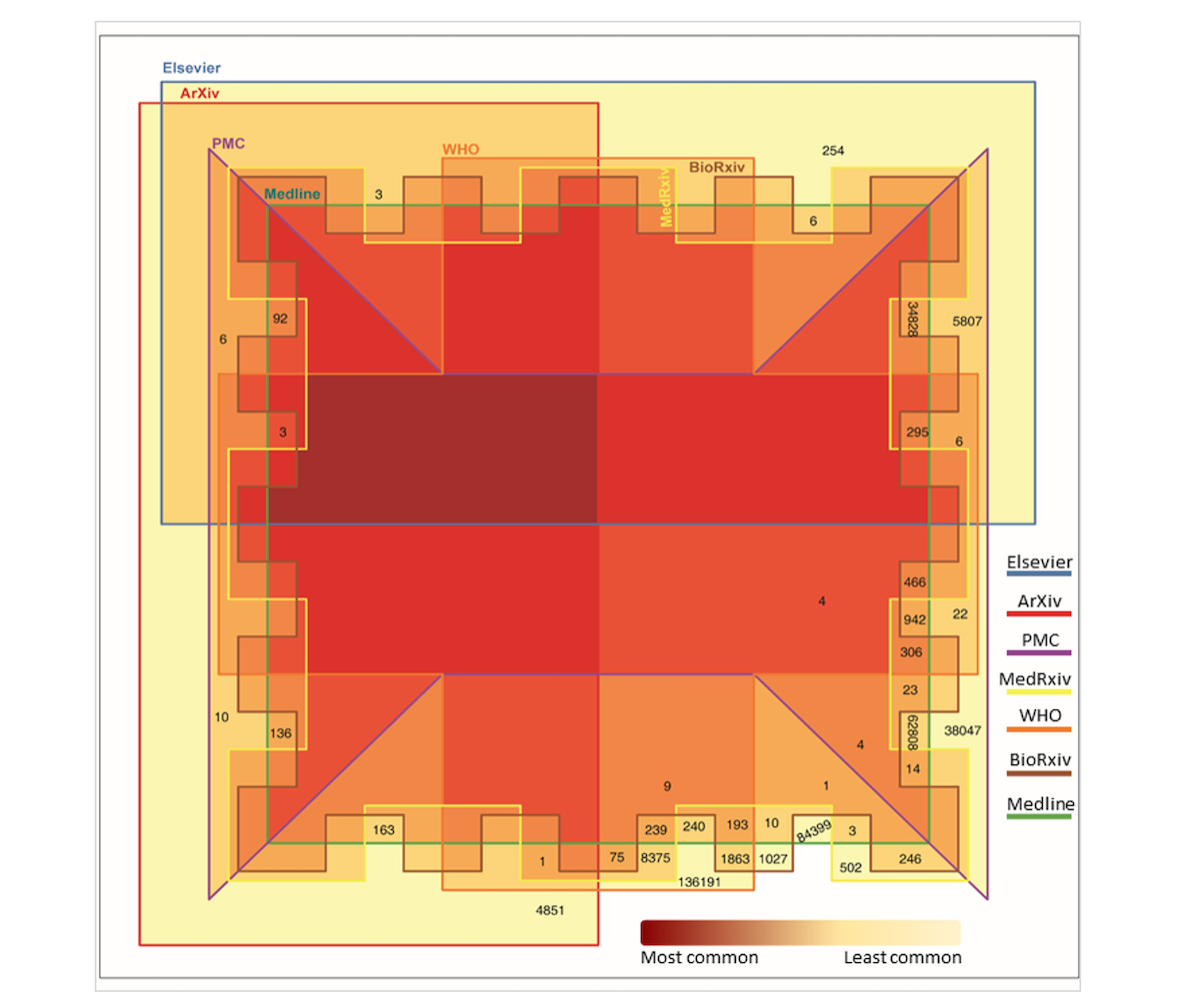
Number of articles published unique to each resource with no intersection (ie, these articles were not published in more than one resource). PMC: PubMed Central, WHO: World Health Organization.

One of the CORD-19 data set features is the article’s source, which represents the name of the publisher. Overall, 7 unique sources are enlisted; however, multiple sources are mentioned for articles published in more than one source. The 7 individual sources comprise articles that are either unique to them or are shared with other sources. In Figure 7, the articles shared among different resources are visualized using a Battle Venn diagram where the common area represents the number of shared articles. The intensity of color shows the high number of sources that share those articles. For instance, Medline has 84,399 articles that are not published anywhere else. However, it shares several articles with other sources; for example, with PMC, it shares 62,808 articles. No article is common in all the sources; however, 4 articles were shared among all the sources except Elsevier and arXiv. The R programming code and data files are available from the “COVID-19-Resource-Categorization” GitHub repository [31].

#### LitCovid

LitCovid is a hub of curated literature of scientific articles about COVID-19 [32-34]. The source is updated daily and has access to about 29,000 (and still growing) articles in PubMed. One unique feature of this effort is that identification of relevant articles is 35% better than the conventional keyword-based searches [34]. For improved accessibility, additional information has been added. The articles are categorized by research topics that include general information, mechanism, treatment, case report, transmission, disease diagnosis, prevention and epidemic forecasting, and geographical locations [34]. Under each topic, three additional important pieces of information are indexed—chemicals (the name of chemical products, like Remdesivir, used in different trials), journals, and countries (the host country of the research).

**Figure 8:**
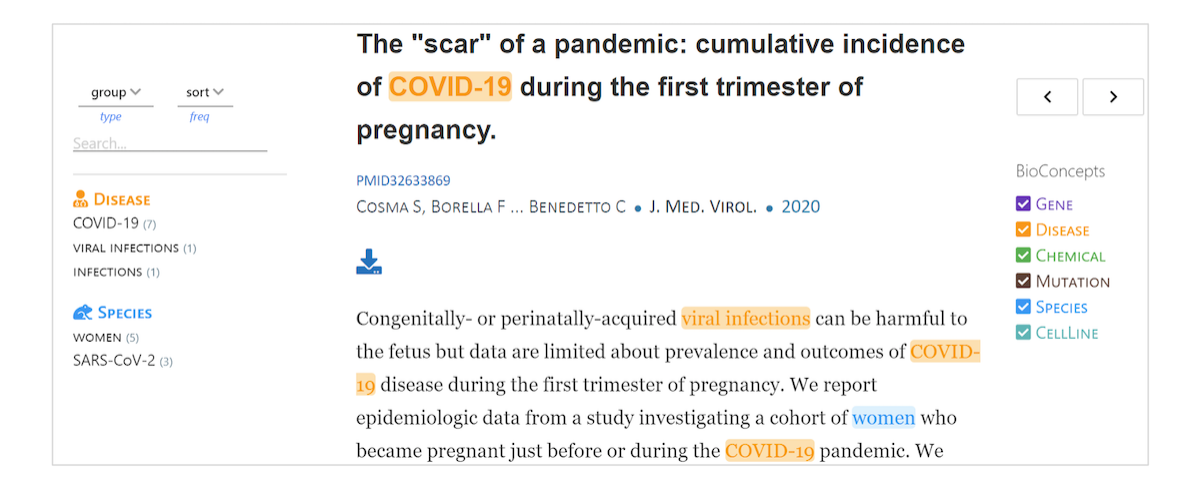
Annotated entity types highlighted in different colors in the title and abstract.

An associated effort extended by PubTator Central [35] derives articles from LitCovid and annotates them with 6 entity types, also called bio concepts—gene, disease, chemical, mutation, species, and cellline. The annotations of these entity types are made in different colors (Figure 8). For example, if a disease concept appears in the title or abstract, it is highlighted in yellow. Similarly, chemical names appear in green and a gene in purple. These annotations support text- and data-mining activities and are available for download in XML (Extensible Markup Language) format.

#### COVID-19 Evidence Alerts

COVID-19 Evidence Alerts is a McMaster University service that alerts users to current best evidence about COVID-19 [36]. It notifies users about the reports published in MEDLINE-based journals, which are critically appraised for scientific merit. Each appraised report is assigned a study category: diagnosis, etiology, treatment, prognosis, guideline, and clinical prediction. Reports with sufficient scientific merit are appraised as “higher-quality studies for clinical attention.” Studies that provide lower-quality scientific evidence are posted as well with at least one reason for their lower-quality designation (eg, not a randomized controlled trial). There are also reports that are relevant but may not be assigned to a specific study category.

This service is beneficial for applications that involve clinical decisions. As of June 30, 2020, about 2700 reports had been posted accumulatively in all three categories; this list is growing and updated every weekday. Out of all the appraised reports, about 70 belong to the higher-quality category; this demonstrates that less than 3% of reports meet the criteria for clinical relevance and scientific merit; 34% did not meet the criteria for scientific merit, and 63% did not belong to a study category. There were two times more studies that did not belong to a study category than those that belonged to at least one study category.

#### CALC-19

The COVID-19 Advanced Literature Classifier (CALC-19) is a classifier of medical literature about COVID-19. The data are updated every week; as of July, the data set included more than 150,000 scholarly articles where each article is tagged by country, year, source, topic, and keywords [37]. CALC-19 extracts all of its articles from the CORD-19 data set and adds metadata like MeSH (Medical Subject Headings) keywords, authors, date of publication, and journal of publication in addition to the title and abstract. Users can search for articles of interest using different filters and tags provided by the service. The searched articles can be downloaded in Excel, CSV, Endnote, RIS, BibTex, and a new Health Level Seven (HL7) Fast Healthcare Interoperability Resources (FHIR) JSON format. The CALC-19 service is powered by the PICO Portal, which provides a platform to accelerate research and innovation by leveraging artificial intelligence and creating efficient systematic reviews of studies.

#### Evidence Aid COVID-19

Evidence Aid [38] is a collection of summaries of high-quality research studies. The collection is available in English with translations in 5 languages: French, Spanish, Portuguese, Arabic, and Chinese. This collection comprises summaries of systematic reviews to provide a quick overview of relevant and impactful information about COVID-19 concerning health conditions, outcomes, and other aspects necessary for the recovery period.

Each summary of a systematic review consists of subsections such as “Citation,” “What is this?” and “What was found.” The citation section includes information on the title of the systematic review, authors, publishing venue, date, and page information. The “What is this?” section summarizes information about population, experimental setup, environment, location, etc. The “What was found” section describes the findings and outcomes of the trials included in the systematic review (eg, the impact of a drug on patients with COVID-19 as positive, negative, or no effect).

#### Comparative Summary of Initiatives

The initiatives discussed as a part of the secondary-level resources are summarized in Table 3 to provide a comparative analysis of different aspects, such as dependent resource information, applications, language support, human validations, and download formats.

**Table 3:** Comparative summary of secondary-level resources based on different features that are important for researchers to expand their work on COVID-19.

Initiative or feature	Baseline resource	Direct applications	Update frequency	Multilingual support	Human expert verified	Export format
CORD-19^a^	Original research	IR and TM^b^	Weekly	N/A^c^	N/A	CSV^d^
LitCovid	PubMed	IR and TM	Daily	N/A	Yes	RIS^e^, TSV^f^
COVID-19 Evidence Alerts	CORD-19	Systematic review	Every weekday	N/A	Yes	N/A
CALC-19^g^	CORD-19	IR and TM	Weekly	N/A	Users can filter records and tags to find records relevant to their work	Excel, CSV, Endnote, RIS, BibTex, HL7 FHIR^h^ JSON^i^
Evidence Aid COVID-19	Systematic review	Evidence-based medicine, clinical decision support, guidelines	N/A	Yes	Yes	N/A

^a^CORD-19: COVID-19 Open Research Dataset.

^b^IR and TM: information retrieval and text mining.

^c^N/A: not applicable.

^d^CSV: comma-separated value.

^e^RIS: Research Information Systems.

^f^TSV: tab-separated value.

^g^CALC-19: COVID-19 Advanced Literature Classifier.

^h^HL7 FHIR: Health Level Seven Fast Healthcare Interoperability Resources

^i^JSON: JavaScript Object Notation.

#### Tertiary Resources

The tertiary-level initiatives use the outcomes of secondary resources for creating clinical guidelines, standards, and vocabularies to provide direct assistance to medical professionals and implementers of clinical decision support systems. The WHO and the CDC are the two comprehensive resources that provide general and all technical guidelines to the public and health professionals. On top of this, other resources add, refine, and customize their findings. The WHO publishes technical guidelines in 14 different categories (eg, clinical care, infection prevention and control, laboratory and diagnosis, etc) [39]. The CDC offers guidelines for various stakeholders; for health workers alone, it provides guidance in terms of 12 categories (eg, testing, clinical care, infection control, etc) [40]. Numerous initiatives derive guidance from the WHO and CDC guidelines, such as Duodecim: EBM Guidelines Coronavirus Infections [41] and COVID-19 guidelines listed in DynaMed [42].

#### COVID-19 Knowledge Accelerator

COVID-19 Knowledge Accelerator (COKA) [43] was first initiated in late March 2020. Led by Brian Alper, COKA is an add-on effort to an ongoing project, Evidence-Based Medicine on FHIR [44], to accelerate the processing of massive research data on COVID-19 in order to summarize and synthesize the evidence in a standard format for computable expression. It aims at resolving inefficiencies in current scientific dissemination systems in which research data are transformed into various noncomputable forms for human displays [45]. This initiative rightly identified the problem area of noncomputable communication and channelized its efforts to construct computable (structured) results directly from research publications, thus accelerating evidence synthesis.

The initiative gained momentum over a short period of time, and as of July 2020, COKA had more than 150 working meetings with more than 40 active participants from more than 25 organizations from academia, industry, government, and nonprofit organizations in 7 countries. Activities under this initiative are divided into 3-team setups: project, process, and system. Under each setup, four workgroup meetings are held every week (for a total of 12 meetings/week). Participants actively contribute to different meetings of their choice every week, and a report of the discussion is shared at the end of each day.

One of the key achievements of this initiative is the development of citation resource schemas and instances. As of July, COKA has created more than 36,000 citation resources for biomedical publications in the CORD-19 data set. Moreover, a profile resource, *EvidenceReport*, is another important outcome of this initiative, an extension of the *Composition* resource. The EvidenceReport resource provides a comprehensive report referring to one or more than one resource(s). As of July, more than 30 example reports related to COVID-10 have been generated. In addition to resource schemas, COKA made tremendous efforts in vocabulary mappings for evidence-related resources. In a short time, COKA developed a 13-step Code System Development Protocol in September 2020. HL7 FHIR is used as the underlying standard to meet interoperability needs and support the global development of terminologies for the exchange of scientific evidence [46].

#### ACTS COVID-19 Guidance-to-Action Collaborative

The AHRQ (Agency for Healthcare Research and Quality) Evidence-Based Care Transformation Support (ACTS) initiative of the COVID-19 Guidance-to-Action Collaborative aims to improve the development, dissemination, and use of “living” COVID-19 guidance [47]. The collaborative supports the COVID-19 “knowledge supply chain,” that is, the data-to-evidence-to-knowledge-to-guidance-to-action sequence to make the processes of guidance development, workflow integration, and knowledge supply chain more efficient and effective. Among its primary functions, the collaborative provides current solutions to urgent clinical challenges faced by health professionals, helps guidance developers in tracking COVID-19–related recommendations, fosters collaboration among implementers of COVID-19 guidance summaries, and facilitates coordination to optimize the flow of COVID-19 “evidence to action.”

The collaborative produces rapid guidance summaries that provide a comprehensive description of existing evidence and guidance from various sources such as the CDC, the WHO, and the European CDC. The guidance summaries are not clinical practice guidelines; therefore, they should not be used or interpreted as such. Instead, they can help develop local recommendations and policies. As of July, 20 guidance summaries have been produced under two categories (ie, patient care and operations). The guidance summary is structured with a question form at the top addressing major recommendations, followed by a list of evidence collected from various sources.

#### National Institutes of Health COVID-19 Treatment Guidelines

In collaboration with other organizations, the National Institutes of Health has developed treatment guidelines to support 297 clinicians in caring for patients with COVID-19 [48]. These guidelines are updated frequently due to the quickly evolving nature of clinical information on the new coronavirus. The guidelines’ recommendations, which are based on scientific evidence and expert opinion, possess two ratings: the strength of the recommendations indicated as a letter (A, B, or C), and the quality of the evidence indicated using a Roman numeral (I, II, or III). A panel composed of experienced representatives from 14 different organizations and societies, such as the American College of Chest and Emergency Physicians, the Food and Drug Administration, and the Society of Critical Care Medicine, has been established to develop these guidelines. The panel utilizes data from the published scientific literature on COVID-19 and the experience of its members to develop the recommendations in these guidelines.

The panel develops recommendations in clinical care areas, that is, care of critically ill patients with COVID-19, including antiviral therapy, immune-based therapy, and adjuvant therapy guidelines for special populations such as pregnant women and children. The panel’s approach to publishing the recommendation in these guidelines can be learned from the example recommendation provided about chloroquine or hydroxychloroquine in the category of antiviral therapy: “The Panel recommends against the use of high-dose chloroquine (600 mg twice daily for ten days) for the treatment of COVID-19 (AI).” This example tells us that the strength of the recommendation is “A,” which means that the statement is a strong recommendation, and the quality of evidence is “I,” which means it is supported with data from one or more randomized trials with clinical outcomes and validated laboratory endpoints.

#### American College of Surgeons Elective Case Triage Guidelines for Surgical Care

The American College of Surgeons (ACS) has developed recommendations for surgeons to identify which procedure should be curtailed [49]. The ACS releases newsletters to update recommendations on curtailing the performance of surgical procedures continuously 2 times a week. As of July, the ACS has published guidelines in 14 categories (eg, cancer surgery, gynecology, neurosurgery, urology, vascular surgery, etc). Most of the guidelines are provided in a descriptive form, but a few include vascular surgery and orthopedic procedures. An example of COVID-19 guidelines for the triage of vascular surgery patients is provided in Table 4, describing the meaning of each tier class (1, 2a, 2b, and 3) concerning surgery postponement.

**Table 4:** Examples of COVID-19 guidelines for the triage of vascular surgery patients.

Category	Condition	Tier class
AAA^a^	Ruptured or symptomatic TAAA^b^ or AAA	3: Do not postpone
Peripheral aneurysm	Asymptomatic peripheral aneurysm	2a: Consider postponing
Bypass graft complications	Revascularization for high-grade restenosis of previous intervention	2b: Postpone if possible
Carotid	Asymptomatic carotid artery stenosis	1: Postpone

^a^AAA: abdominal aortic aneurysm.

^b^TAAA: thoracoabdominal aortic aneurysm.

#### COVID-19–SNOMED Clinical Terms

SNOMED International, a leading health care terminology organization, took steps to identify codes for different terms related to COVID-19 [50]. In the March 2020 interim release, 24 records had been added, which increased to 49 records as of August 11, 2020. A complete list of these concepts with Uniform Resource Identifiers, fully specified name, and preferred term is now available on Confluence [51]. The two most important terms, “SARS-CoV-2” (organism) and “COVID-19” (disorder) have been given the identifiers 840533007 and 840539006, respectively. A map of SNOMED Clinical Terms (SNOMED CT) to ICD-10 (International Classification of Diseases, Tenth Revision) is provided for two concepts: “COVID-19” and “Exposure to SARS-CoV-2,” as described in Table 5.

**Table 5:** SNOMED CT (SNOMED Clinical Terms) to ICD-10 (International Classification of Diseases, Tenth Revision) map.

Preferred term	Source SNOMED CT identifier	Target ICD-10 identifier
COVID-19	840539006	U07.1
Exposure to SARS-CoV-2	840546002	Z20.8

#### Summary of Tertiary Resources

This section provides an inexhaustive list of initiatives that have contributed to the creation of tertiary resources. As shown in Table 6, the initiatives are categorized into three categories: standard, guidelines, and terminologies. Information on the group or organization has been provided to support future applications to catalogue these resources in their services.

**Table 6:** Summary of tertiary resources related to COVID-19–based standards, guidelines, and terminologies.

Category and organization/group		Initiatives
**Standards**		
	Health Level Seven (HL7)	HL7 is a health information exchange standard. Using its current standard—Fast Healthcare Interoperability Resources (FHIR)—different initiatives have been taken place related to COVID-19 that include COVID-19 Knowledge Accelerator (COKA) [43] and the Situational Awareness for Novel Epidemic Response (SANER) Project using HL7 FHIR to enable easier reporting for public health [52].
	International Organization for Standardization (ISO)	The ISO has compiled a list of freely available standards to support global efforts in dealing with the COVID-19 crisis [53]. It also features a list of national resources developed by ISO members in different countries to support the fight against COVID-19 [54].
	American National Standards Institute (ANSI)	In response to the COVID-19 pandemic, the ANSI has initiated the Standards Alliance Phase 2 (SA2) [55], which aims to reduce importation and regulatory barriers of COVID-19 testing kits and training resources on the use of medical devices and testing equipment.
**Guidelines and recommendations**		
	National Institutes of Health (NIH)	The NIH has developed a comprehensive set of COVID-19 treatment guidelines [48].
	American College of Surgeons (ACS)	The ACS Elective Case Triage Guidelines for Surgical Care [49] aims to develop recommendations to help surgeons identify which procedures should be curtailed.
	World Health Organization (WHO)	The WHO is perhaps the largest source of information, guidance, and recommendations to support the fight against COVID-19. In addition to general public health recommendations, it provides technical guidance on different topics [39].
	Centers for Disease Control and Prevention (CDC)	Like the WHO, the CDC offers advice, recommendations, and guidelines for different stakeholders. For health workers alone, it provides guidance on 12 categories that include testing, clinical care, infection control, etc [37].
	American Gastroenterological Association (AGA)	The AGA has developed recommendations based on the systematic review and meta-analysis of 47 studies and 10,890 unique patients with gastrointestinal symptoms [56].
	US Food and Drug Administration (FDA)	The FDA provides guidance related to drug development programs and the food industry impacted by COVID-19, such as donating COVID-19 plasma and facilitating the development and availability of medical device therapeutics to combat COVID-19 [57].
**Vocabularies and terminologies**		
	SNOMED Clinical Terms (SNOMED CT)	As of August 11, 2020, SNOMED International has added 49 records related to COVID-19 [50].
	International Classification of Diseases, Tenth Revision, Clinical Modification (ICD-10-CM)	The ICD-10-CM is an official coding and reporting guideline that provides important information needed to understand the usage of ICD codes in the context of COVID-19 [58]. These codes can be searched in a newly released user-friendly browser [59].
	Unified Modeling Language System (UMLS)	The UMLS provides a set of COVID-19–related terms [60] mostly mapped to SNOMED CT and Medical Subject Headings.
	Medical Subject Headings (MeSH)	A new MeSH Supplementary Concept Record (SCR Class 3-Disease) was added on February 13, 2020, to the 2020 MeSH Browser in response to COVID-19 [61]. The most current updates are found on the MeSH Browser [62]. Using MeSH terms, the recommended search strategy for retrieving COVID-19–related biomedical studies is “2019-nCoV OR 2019nCoV OR COVID-19 OR SARS-CoV-2 OR ((Wuhan AND coronavirus) AND 2019/12[PDAT]:2030[PDAT]).”
	Logical Observation Identifiers Names and Codes (LOINC)	LOINC introduced 264 codes in response to COVID-19 [63]. These codes are distributed in 5 categories: SARS-CoV-2 lab tests (84 terms), LOINC terms for SARS-CoV-2 ask-at-order-entry questions (9 terms), convalescent plasma (2 terms), LOINC terms related to public health case reporting (63 terms), and COVID-19 or telehealth documents (106 terms).

#### COVID-19 Dashboard Applications

An array of dashboards has been proposed and implemented to portray the influx of data and information related to COVID-19. Here, we first discuss a set of well-known dashboards in use and provide information on different forms that include structured, unstructured, plane, and graphical.

### Johns Hopkins University Interactive COVID-19 Dashboard

In response to the COVID-19 emergency, the CSSE at Johns Hopkins University (JHU), in the United States, developed an online interactive dashboard to visualize and track coronavirus cases in real time [51,64]. Starting on January 22, 2020, the dashboard data were updated manually, but with the increase in the number of cases, the manual reporting process became unsustainable and a semiautomated living data stream strategy has been used since February 1. This dashboard relies on several data sources (eg, for the identification of new cases) and seeks data from DXY China (it was initially the only data source), Twitter feeds, online news, and direct communication sent to the dashboard. The case numbers are duly confirmed with regional and local health departments, including the respective CDCs, health departments, and the WHO. This dashboard’s main feature is real-time interactivity, which enables users to see cumulative cases, active cases, and other information like incidence rate on a global map. Users can click and select a region on the map and see that region’s statistics. A US map is provided separately with county-wise confirmed cases and deaths. Moreover, it supports data in motion that show daily trends of cases and deaths in different regions.

### WHO COVID-19 Dashboard

Like the JHU CSSE interactive dashboard, the WHO provides a live dashboard with COVID-19 case numbers and deaths where users can use their mouse to hover over a map to obtain an overview of cases across the world [16]. It provides two kinds of maps: a bubble map and a choropleth map. Alongside the maps are data tables detailing country-wise cumulative confirmed cases, deaths, as well as newly reported cases and deaths in the last 24 hours. It facilitates region-wise searches (eg, Europe, Asia, and Africa) as well.

### Worldometer Coronavirus Updates

Run by an international team of developers, researchers, and volunteers, Worldometer provides global COVID-19 real-time statistics on data collected, analyzed, and validated from thousands of sources worldwide [65]. The data are claimed to be trusted and used by different governments (eg, the United Kingdom, Thailand, Pakistan, Sri Lanka, Vietnam, etc) and private organizations (eg, JHU CSSE, the BBC, the New York Times, etc). It provides country-wise statistics of new cases, deaths, recovered cases, critical cases, tests per million, and other important information. The most informative feature is the searchable and clickable country-wise data table that can be customized to include or exclude columns such as new cases, total cases, deaths, and tests per million, which are presented along with the total population of that country.

#### Other Efforts

Other than the dashboards mentioned above, dozens of dashboards are available for use scoped by a specific country, territory, or region. Coronaboard [66] provides COVID-19 statistics globally for the United States and three other countries (the Netherlands, France, and South Korea) in their respective languages. Almost all countries affected by the COVID-19 pandemic have put forth efforts to keep their populace informed via easy-to-use and understandable communication methods. For instance, Japan has developed its own dashboard [67], which supports the Japanese language and other languages such as English, German, French, and Arabic. Moreover, different media groups such as CNN, the BBC, and the New York Times have devised sophisticated dashboards, which rely heavily on JHU data.

#### A Conceptual Framework COVID-19–Related Knowledge Resources

**Figure 9:**
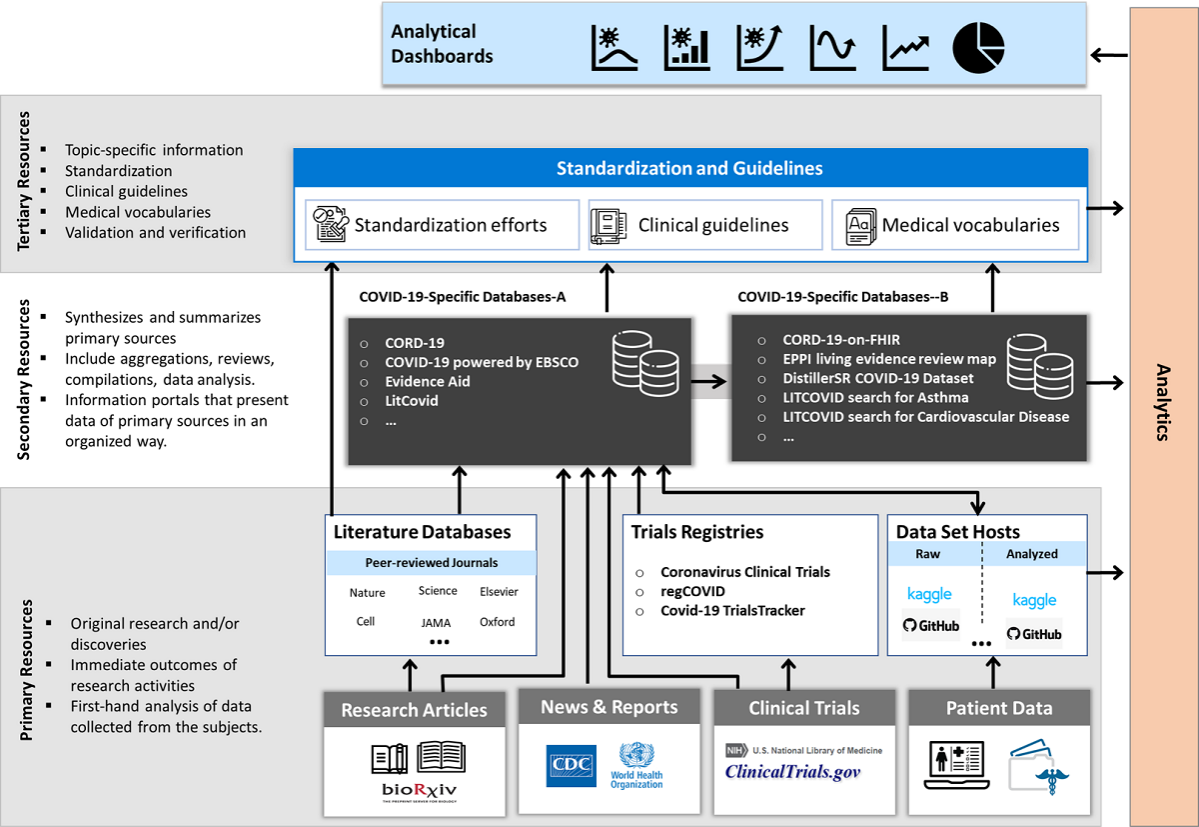
An enterprise architecture framework for COVID-19–related efforts and initiatives involving primary, secondary, and tertiary knowledge resources. CORD-19: COVID-19 Open Research Dataset, FHIR: Fast Healthcare Interoperability Resources, EPPI: Evidence for Policy and Practice Information and Co-ordinating Centre, JAMA: Journal of the American Medical Association.

Investigating diverse information at the three levels discussed earlier enabled us to develop an enterprise architecture framework for COVID-19–related knowledge resources. As illustrated in Figure 9, the framework comprises the categorization of efforts and initiatives, and organizes resources at the primary, secondary, and tertiary levels, demonstrating resource interconnectedness and flow of information.

The framework’s key feature is to enable tracking of a context in terms of information resources at different levels. For instance, if a user is interested in information that is an outcome of a secondary-level initiative, the dashboard will provide the required information resource(s) along with the dependent resource(s) at the primary level. The resources are then visualized in the form of dependency graphs generated automatically in response to the user query.

Figure 9 is a high-level conceptual illustration of the organization of COVID-19 resources in three logical layers connected to analytics that enable the creation of user applications such as analytical dashboards. In the following sections, we discuss the physical design of resources at different levels with relationships and properties, which demonstrates the approach’s practicality.

#### Conceptual Modeling Using Knowledge Graphs

**Figure 10:**
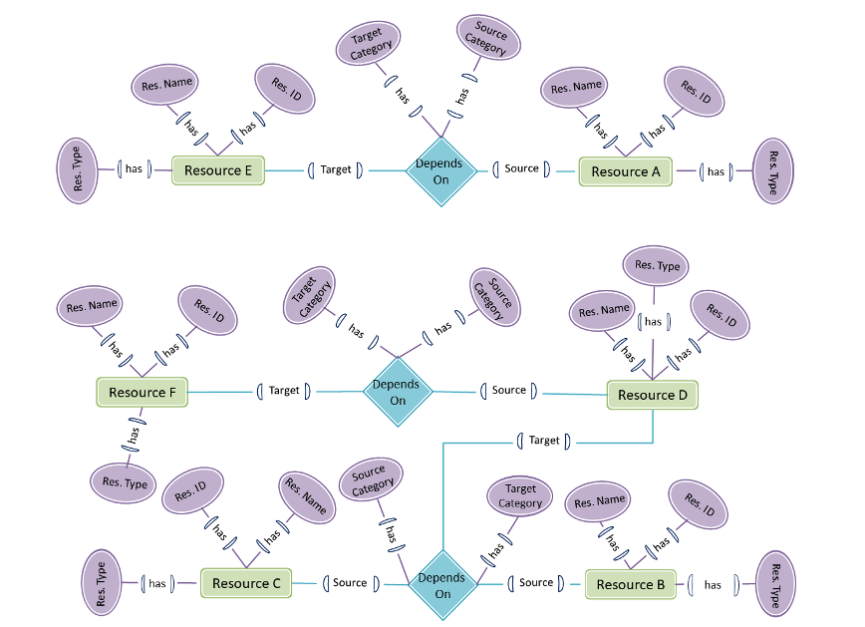
A knowledge graph representation reflecting an example scenario of 5 resources A, B, C, D, and E with their mutual dependencies.

A robust and semantically enriched model is needed to manage the highly interlinked COVID-19 information resources and metadata. Based on the proposed framework, we created a knowledge graph design to represent interlinked resources to put data in context via linking and semantic information. An example scenario is presented in Figure 10 to display the suitability of the knowledge graph. Let us have 6 resources named A, B, C, D, E, and F, where three resources (A, B, and C) belong to primary, two resources (D and E) to secondary, and one (F) to tertiary. Let resource F be dependent on resource D, which depends on resources B and C. Similarly, let resource E be dependent on resource A. The corresponding nodes in the knowledge graph to represent the dependency among resources is shown using the relationship node *depends on*, which has two attributes, *source category* and *target category*. The source refers to the category of resources it depends on, and the target refers to the category of resource created as a result.

Each resource has at least three attributes—identifiers, name, and type—where the type represents the subcategory within the primary, secondary, or tertiary category. For instance, resources whose type is data resources are research publication resources in the primary category.

#### Maintenance of Knowledge Graphs

The rich feature set of knowledge graphs that include classes, relationship types, and categorization support greatly help to resolve the resource maintenance challenge. Adding a new resource to the database by assigning the correct class of primary, secondary, and tertiary requires the users to input metadata about the resource. The input data may consist of dichotomous questions to support structured queries run on the underlying graph database to infer the target class for the resource, and saving a new resource must look for duplication by checking multiple parameters.

#### Visualization Using Knowledge Graphs

Current COVID-19 dashboards typically represent time-series visuals and geographic maps, with exceptions of dashboards that respond to the pandemic by showing clinical trials, policy- and finance-related interventions, and social distancing directives [68]. Some dashboards include contact tracing data; however, resource dependency–tracking dashboards are not seen in the literature. Novel knowledge graph visualization algorithms are emerging, customized to support resource tracking with their dependencies and metadata semantics.

## Discussion

We conducted this study to classify COVID-19 resources into a three-level structure. We designed a two-phase research design approach to locate COVID-19 resources and their placement, and conceptualize the architecture in the first phase while developing and testing the contextual dashboards in the second phase. We surveyed various efforts and initiatives worldwide, provided descriptive statistics, and classified them into primary, secondary, and tertiary categories. The proposed categorization led us to design knowledge graph–based models for developing contextual dashboards. A dependency graph theory was incorporated to visualize the results of information resources and their interdependence. The proposed work enables other applications such as search engines, interactive dashboards, and tracking systems to capture contextual information of COVID-19–related resources. Using knowledge graph models for application development will add transparency to the information infrastructure, thus increasing the trust factor. Moreover, the semantics captured in subgraphs of the whole COVID-19 resource categorization knowledge graph can provide more domain information and thus significantly improve the performance of machine and deep learning models [69].

The knowledge graph has the capabilities to store a resource and associated metadata about the resource. The metadata is linked to the resources via a well-established relationship mechanism defined in the knowledge graph. These relationships provide a baseline context to the stored resources, making them more explicit and distinctive from each other. Using existing relationships in the knowledge graph could create new types of relations in the knowledge graph. Using reasoning on these extended contextual relationships could help recognize and classify the COVID-19 resources into three explicit categories. In addition, we can seek help from human experts to create an initial set of resources with explicit categorization, which can be used as a seed input to develop a machine learning model. The learned machine learning model can be used as a classification model for resource categorization, and the feature set can be used to create entities and relationships in the knowledge graph.

Moreover, knowledge graph–based representation will be a great source of assistance in data preparation for machine learning models. For instance, the source and target entities associated with each other through a relation communicate a context that is useful in word embeddings. Using machine learning over other graph databases lacks two main components: (1) the metadata related to data, which is coined as lacking a piece of contextual information; and (2) the lack of a functional module, also called the reasoning module, to interpret or transform the actual data in the presence of metadata. The knowledge graph as a source to machine learning exposes the actual data, metadata, and contextual relationships. The additional meta information and contextual relationship improve machine learning outcomes in three main ways. First, the model created from actual data associated with contextual information provides more intelligent predictions or accurate classification. Second, based on the learning patterns of machine learning, the knowledge graph reasoning capability enhances the model with additional rules and generates other contextual relationships. Third, if required (and this depends on the design of the knowledge graph), some information may be inferred by the knowledge graph during modeling and embedded to the learning phase of the machine learning method.

This study’s resource categorization can help develop applications for various purposes for combatting the novel coronavirus. First, it provides a baseline for the further categorization of resources in each level. Further classifications can be introduced in each level to make its use more specialized and customized. Second, it allows for the development of customized search engines for users to obtain results more precisely. Third, it enables specialized dashboards constructed on the information to be structured into three levels. Finally, the proposed framework is extendable to bring clinical and genomic resources together by highlighting their associations to help disease monitoring and tracking.

## Abbreviations

ACM: Association for Computing Machinery
ACS: American College of Surgeons
ACTS: AHRQ Evidence-Based Care Transformation Support
AHRQ: Agency for Healthcare Research and Quality
CALC-19: COVID-19 Advanced Literature Classifier
CDC: Centers for Disease Control and Prevention
chiCTR: Chinese Clinical Trials Registry
COKA: COVID-19 Knowledge Accelerator
CORD-19: COVID-19 Open Research Dataset
CSSE: Center for Systems Science and Engineering
CSV: comma-separated value
FHIR: Fast Healthcare Interoperability Resources
HL7: Health Level Seven
ICD-10: International Classification of Diseases, Tenth Revision
ICTRP: WHO International Clinical Trials Registry Platform
ISRCTN: International Standard Randomised Controlled Trial Number Registry
JAMA: Journal of the American Medical Association
JHU: Johns Hopkins University
JSON: JavaScript Object Notation
MeSH: Medical Subject Headings
NEJM: New England Journal of Medicine
PMC: PubMed Central
SNOMED CT: SNOMED Clinical Terms
WHO: World Health Organization
XML: Extensible Markup Language
